# Fundamental Frequency Contour (Melody) of Infant Vocalizations across the First Year

**DOI:** 10.1159/000528732

**Published:** 2022-12-20

**Authors:** Tabea Kottmann, Maren Wanner, Kathleen Wermke

**Affiliations:** Center for Pre-Speech Development and Developmental Disorders, University Hospital Würzburg, Würzburg, Germany

**Keywords:** Infant, Crying, Babbling, Melody, Vocal development

## Abstract

**Introduction:**

The fundamental frequency contour (melody) of cry and non-cry utterances becomes more complex with age. However, there is a lack of longitudinal analyses of melody development during the first year of life.

**Objective:**

The aim of the study was to longitudinally analyze melody development in typical vocalization types across the first 12 months of life. The aim was twofold: (1) to answer the question whether melody becomes more complex in all vocalization types with age and (2) to characterize complex patterns in more detail.

**Methods:**

Repeatedly recorded vocalizations (*n* = 10,988) of 10 healthy infants (6 female) over their first year of life were analyzed using frequency spectrograms and fundamental frequency (f0) analyses (PRAAT). Melody complexity analysis was performed using specific in-lab software (CDAP, pw-project) in a final subset of 9,237 utterances that contained noise-free, undisturbed contours. Generalized mixed linear models were used to analyze age and vocalization type effects on melody complexity.

**Results:**

The vocalization repertoire showed a higher proportion of complex melodies from the second month onward. The age effect was significant, but no difference was found in melody complexity between cry and non-cry vocalizations across the first 6 months. From month 7–12, there was a further significant increase in complex structures only in canonical babbling not in marginal babbling. Melody segmentations by laryngeal constrictions prevailed among complex shapes.

**Conclusion:**

The study demonstrated the regularity of melody development in different vocalization types throughout the first year of life. In terms of prosodic features of infant sounds, melody contour is of primary importance, and further studies are required that also include infants at risk for language development.

## Introduction

Infants are not yet competent communication partners at birth, but they are already able to perceive auditory information of their surrounding world based on melodic cues [1, 2, 3, 4]. They mainly perceive the time-varying fundamental frequency (f0), i.e., the melody, which is the most salient acoustic cue for young infants [5, 6]. The aptitude and inborn intention to implement perceptive experience in vocal production start shortly after birth, when neonatal crying is shaped by speech melody of the surrounding language [7, 8, 9]. These imprints are traces of the early maturity of the auditory system. The acquisition of speech-specific prosody during vocal development, in which melody plays a crucial role, is based on a universal developmental program that is influenced by neurophysiological maturation and learning processes.

Based on many years of research experience in their labs, Wermke and Mende have summarized melody development in pre-speech utterances in a melody development (MD) model [10, 11, 12]. The MD model expresses early vocal development by an ordered sequence from initially prevailing simple (single-arc) melody contours to more and more complex (multiple-arc) contours. Furthermore, this complexity pattern is assumed to be regular, unidirectional for each vocalization type, and universal in nature [12]. According to Wermke and Mende, the MD model describes a “simple” pre-speech melody as a melody consisting of an arc-like ascending-then-descending contour of the fundamental frequency (f0). Four shape prototypes were commonly described: (1) a fast-ascending then slowly descending arc ≙ a left-accentuated melody type, (2) a slowly ascending then fast-descending arc ≙ a right-accentuated melody type, (3) an almost symmetrical ascending-then-descending arc ≙ a symmetrical melody type, and (4) a flat melody at a certain frequency ≙ a plateau type. In contrast, a “complex” pre-speech melody consists of two or more concatenated single arcs. Hence, the single melody arc is interpreted as the most basic unit of melody contour, i.e., a basic building block. The MD model further claims that the basic building block has different shape prototypes which are combinable in any possible sequence, only constrained by physiological reasons. The observation that melody development in pre-speech utterances is characterized by a systematic increase in melody complexity over the first few months seems to reflect a general evolutionary principle for composing complex hierarchical structures [10, 11, 12].

A recent study [13] corroborated the MD model by demonstrating a continuous progression from simple to complex melodies that were evident in both cry and non-cry vocalizations across the first 6 months of life. Simple rising-falling, i.e., single-arc melody contours, were differentiated from complex, multiple-arc contours. Non-cry utterances comprised several vocalization types, e.g., vocants, coos, babbling (ibid.).

The existence of simple and complex melody structure has also been reported in earlier work on vocal development [14, 15, 16]. Simple, single-arc melody types were commonly assigned to “rising,” “falling,” or “flat” types [17, 18, 19, 20]. These melody types were analyzed mainly in infant crying, often pain cries, as it was observed that they could have a diagnostic valence in the context of early detection of central nervous system dysfunction (cf. review in [21]). In research outside pediatrics on sound production in infants, however, melody and its shape received little attention in previous vocal models [22, 23, 24, 25, 26, 27] as the focus was put on the emerging “speech capacity.” In the search for “speech-like” elements in pre-speech vocal sounds, the melody was often forgotten and the occurrence of consonant- and vowel-like elements, syllable quality, or voice features have been the central aspect. However, there is general agreement that language development starts with prosody and hence, melody development requires the same attention as segmental milestones in adequate models of vocal development. In contrast to older models, the MD model puts melody development in the center of early vocal development and also claims its function as a scaffold for the emerging articulatory development [28, 29]. Precursors of segmental properties (vocants, closants) are initially rehearsed on simple, single-arc melodies before full vowels and consonants are intentionally produced in utterances with complex melody contours [13]. However, a systematic study of melody development from simple to complex contours under ongoing maturation of the articulatory system is still pending, particularly during the second half of the first year of life.

For the first time, the present paper aimed to investigate how melody development is accomplished in individual infants longitudinally from birth to 12 months of life. Previous studies have mainly investigated natural crying, while longitudinal studies of melody development in cry and non-cry vocalizations have been lacking in individual infants across the first year of life. The novelty of the present study is its comprehensive objective investigation and description of the age-dependent unfolding of melody patterns within different vocalization types in the same infants. As far as we know, this has not been done yet.

## Subjects and Methods

### Participants and Data Sets

Audio files of healthy, term-born (≥37 gestational weeks) monolingual German infants were analyzed from birth to the age of 373 days of life. Inclusion criteria were an inconspicuous pregnancy, age-appropriate somatic development at birth, an inconspicuous perinatal adjustment (APGAR score of ≥8 at 5 min and ≥9 at 10 min), metabolic indices in the normal range, and passing the newborn hearing screening, i.e., healthy infants. The mean birth weight was 3,201 g (range 2,710–3,935 g); mean head circumference, 35 cm (range 31–37 cm); and mean birth length, 51 cm (range 47–55 cm). The mean gestational age was 39 weeks (range 37–41 weeks). Based on the archived files, we were able to confirm that none of the children showed any developmental delay or disorder in terms of linguistic or cognitive development during the first 3 years of life.

For this longitudinal study, the vocalizations of 10 (6 female) healthy infants were selected. Here, we used all cry and non-cry vocalizations (*n* = 10,988) available in the archive from our participants across the observation age. A vocalization was defined as the total vocal output during a single expiration.

The anonymized audio files came from the sound archive at the Centre for Prespeech Development and Developmental Disorders at the University Hospital of Würzburg. The archive contains anonymized audio files (wav format) of the original recording sessions (sequences of cry, non-cry, and babbling vocalizations) as well as all the individual sounds, which were previously manually segmented using a commercially available system (CSL 4500; KayPENTAX, USA). Finally, all recordings were archived as anonymized data sets. Each parent had a minimum of high school education, and the monthly family income was reflective of a middle-class standard of living.

Cry vocalizations (spontaneous, naturally occurring crying) were recorded under comparable conditions in a hospital (first week) and at home, respectively (e.g., before breastfeeding, relaxed, pain-free manner). As outlined in more detail elsewhere [13], non-cry vocalizations and advanced babbling (marginal and canonical babbling) were recorded in infants' homes during joyful mother-infant interactions. All vocalizations were spontaneously uttered by the infants, and any elicitation of vocalizing was avoided. The length of an individual recording session ranged from about 1 to 3 min (crying) and 1 to 30 min (non-cry and babbling recordings).

Based on visual inspections of the spectrograms (CSL 4500; KayPENTAX, USA), phonatory noise phenomena (episodes of low-dimensional deterministic chaos [30]) and phenomena like sudden fundamental frequency (f0) shifts or highly unstable f0 contours were identified. Vocalizations containing such features were excluded from melody pattern analysis by assigning them as “no-pattern.” Reliable melody analysis would not have been possible in these cases. Very short vocalizations (≤300 ms) were also excluded because the melody cannot unfold properly in the short time available. Excluding these vocalizations (*n* = 1,751; 16%), the finally available database totaled 9,237 vocalizations; comprising of 1,141 spontaneous cry utterances (3–195 days of life) and 4,667 early non-cry vocalizations (4–200 days) recorded across the first 6 months of life. Further data comprised recordings between 7 and 12 months of 3,429 babbling utterances (181–373 days). Note that the term “non-cry vocalizations” comprised vocants, coos, and primitive, mainly nonsyllabic babbling. A differentiation of the babbling utterances in marginal (*n* = 2,754) and canonical (*n* = 675) babbling was only performed between month 7–12 of life. However, all infants appeared in all (cry, non-cry, marginal, canonical babbling) data sets. Table 1 reports the vocalizations analyzed per child per month.

### Vocalization Type

**Fig. 1:**
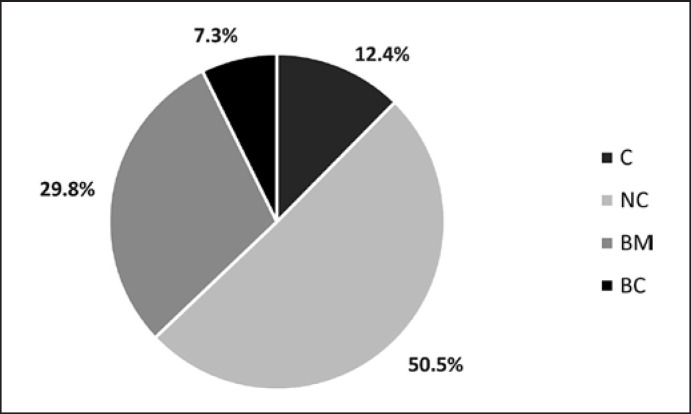
Proportion of individual vocalization types identified in the total repertoire of all 10 infants across the observation period.

Assignment of vocalizations to a certain vocalization type was based on spectrograms and auditory impression. According to the guidelines for coding pre-speech utterances, force choice approach was applied to differentiate between cry, non-cry, and advanced babbling, respectively [23]. Four vocalization types were differentiated: (1) crying, (2) primitive non-cry, (3) marginal babbling, and (4) canonical babbling (Fig. 1). For reasons of reliability, the vocalization types (3) and (4) were only differentiated between 7 and 12 months, i.e., all babbling in the age period before was comprised in the non-cry category (NC). The distinction between marginal and canonical babbling was done by applying the criteria postulated by Buder et al. [23]. However, canonical babbling was not verified by broadband spectrograms in all cases; the assignment was mainly based on auditory impression of “well-formedness” (cf. [22, 23]). This seemed sufficient in most cases since two of the authors are musicians having a trained ear and reached a high agreement in the assignment of the vocalization types (>91%). However, in ambiguous cases, broadband spectrograms were analyzed to identify canonical babbling.

### Melody Analysis

As elsewhere described in more detail [13], an automatic f0 measurement (melody contour analysis) was performed for all 9,237 vocalizations using PRAAT v. 6.0.387. A post-processing verification included removal of high-frequency modulation noise and artifacts. In cases of obvious f0-tracking problems of the automatic routine, f0 determination was manually repeated by two of the authors (T.K., M.W.) using PRAAT. Using the f0 data calculated with PRAAT, melody pattern analysis was performed using specific in-lab software (CDAP, pw-project) which was implemented as a routine procedure at the Center for Prespeech Development and Developmental Disorders. Using CDAP, melody diagrams with a logarithmic scale for f0 and a quarter-tone grid were drawn, and a low-pass filter (Gaussian filtering) was applied with a cutoff frequency of about 40 Hz to eliminate high-frequency modulation noise and artifacts [28].

### Melody Pattern Analysis

**Fig. 2:**
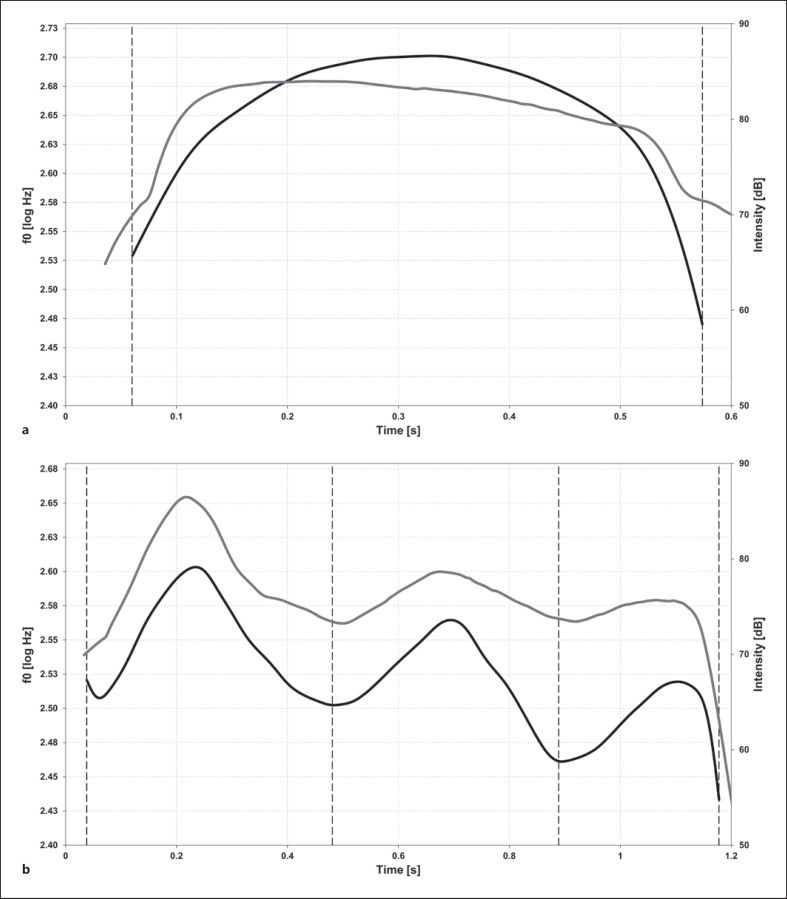
**a, b** Melody/intensity diagrams. Melody (black) and intensity (gray) curve of a single-arc melody (**a**) and complex, triple-arc melody (**b**).

In agreement with preceding studies, the analysis of melody pattern was based on a differentiation between simple (singe-arc) and complex melodies (Fig. 2a, b). Complex melodies were defined as exhibiting ≥ two arcs and/or intra-melodic breaks between arcs by laryngeal constrictions that generate rhythmical variations [11, 31]. Preceding studies defined a melody arc as being longer than 150 ms and as exhibiting a frequency modulation amplitude (FM amplitude) of at least three (cry) or two (non-cry) semitones [12]. However, we applied a stronger time criterion and adjusted the FM amplitude criteria for arc definition to vocalization type: arc duration was defined to require more than 300 ms and a minimum FM amplitude of two (cry), one and a half semitones (non-cry), or of one semitone (marginal and canonical babbling), respectively. This distinction was justified because Wermke and Mende [12] characterized melody arcs using a mathematical modeling approach and reported a decreasing FM amplitude from crying toward babbling of six to eleven percent, i.e., one up to two semitones. The criteria defined here took this into account.

**Fig. 3:**
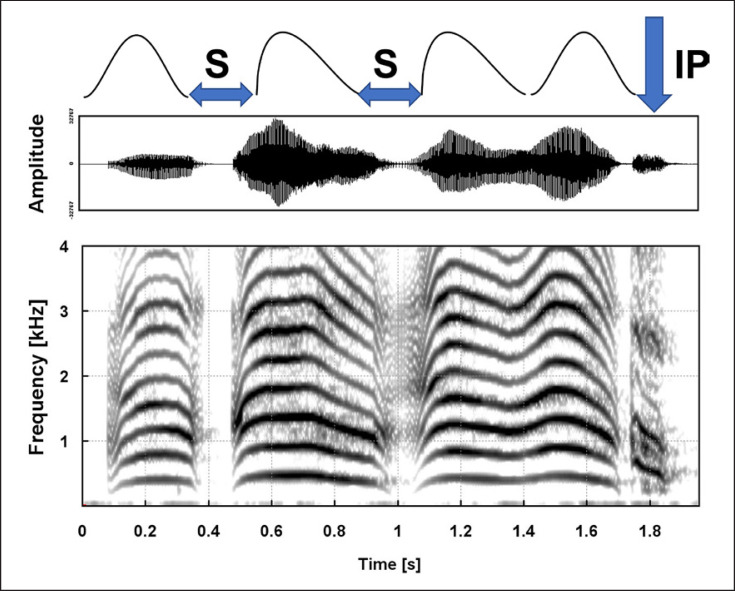
Time waveform and narrow-band (45 Hz) spectrogram displaying a cry utterance consisting of a complex, segmented melody with the following structure: single arc (symmetric shape) − laryngeal constriction (segmentation pause S) − single arc (falling shape) − laryngeal constriction (incomplete segmentation S) − double arc (combination falling-symmetric arc). The subsequent inspiratory noise (ingressive phonation IP) is also visible.

Based on the mentioned arc criteria, all vocalization melodies were objectively subdivided into those with only a simple (single-arc, SA) melody and those with a complex (multiple-arc, MA) melody (cf. Fig. 2a, b). SA contained a single ascending-then-descending melody arc with a minimum duration of 300 ms. Complex melodies consisted of two (DA), three (TA), or more (MA) melody arcs, each arc having a duration of at least 300 ms. Following earlier work [11, 29], we also identified rhythmically segmented contours (SEG) which contain inner-melodic pauses caused by laryngeal constrictions [31]. Complex melodies of pre-speech utterances regularly exhibit rhythmical variations generated by short “segmentation pauses” between adjacent melody arcs across the first year of life (Fig. 3).

Melodies containing at least one complete or incomplete laryngeal constrictions [11, 31] were assigned to “segmented” complex contours (SEG) independent of arc numbers.

### Outcome Measures

The analysis focused on two outcome measures: (1) melody complexity: the melody contour of each vocalization was assigned to one of two categories: (1) “simple,” single-arc melody or (2) “complex,” multiple-arc melody. The percentage of these categories was analyzed across the observation age for each vocalization type.

(2) Complexity degree: To describe the degree of complexity, melody contours of each vocalization type were divided into double-arc (DA), triple-arc (TA), multiple-arc (MA), or segmented structures (SEG) and their relative occurrence displayed in developmental diagrams.

### Statistical Analysis

**Fig. 4:**
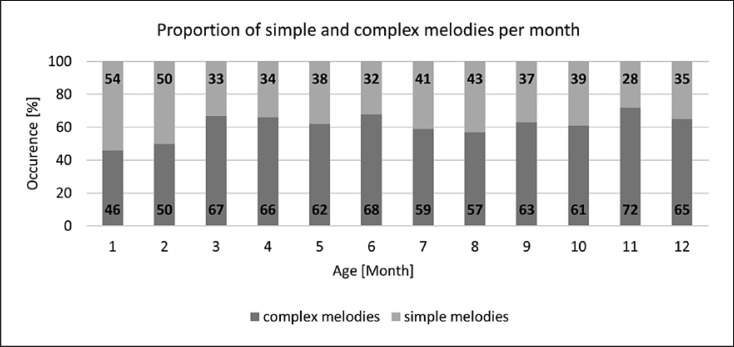
Age-dependent frequency of occurrence of melodies with simple or complex melody.

**Fig. 5:**
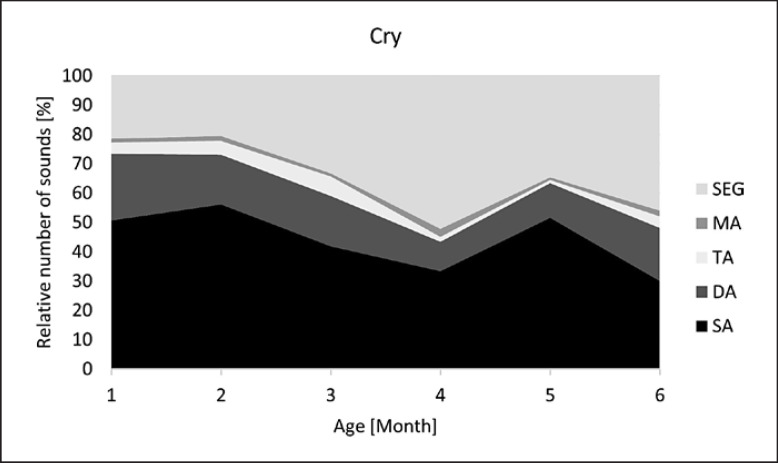
Developmental profile of melody structure in spontaneous, natural crying. Melody structures: SA, single-arc melodies; DA, double-arc melodies; TA, triple-arc melodies; MA, multiple-arc melodies, >3 arcs; SEG, segmentations.

**Fig. 6:**
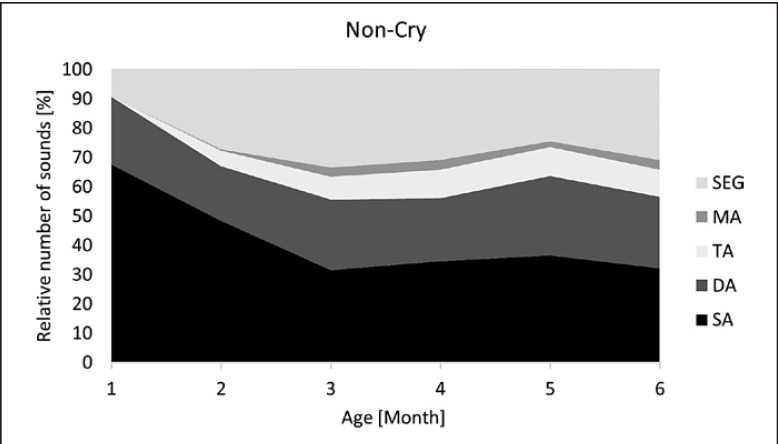
Developmental profile of melody structure in early non-cry vocalizations. Melody structures: SA, single-arc melodies; DA, double-arc melodies; TA, triple-arc melodies; MA, multiple-arc melodies, >3 arcs; SEG, segmentations.

**Fig. 7:**
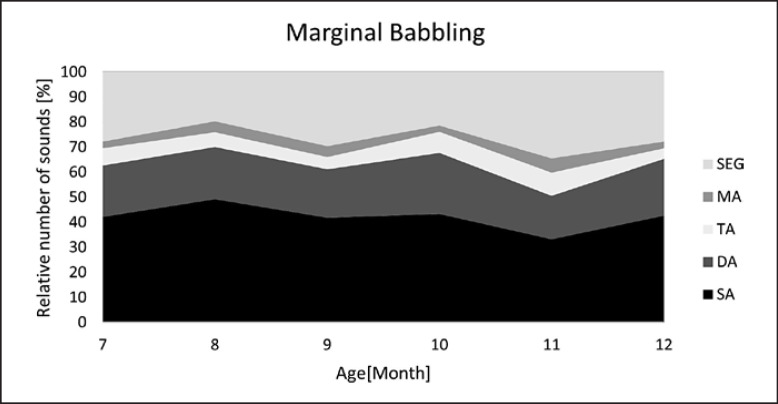
Developmental profile of melody structure in marginal babbling. Melody structures: SA, single-arc melodies; DA, double-arc melodies; TA, triple-arc melodies; MA, multiple-arc melodies, >3 arcs; SEG, segmentations.

Descriptive information about the different melody structures is presented. Pie charts, column charts, and line charts were used for graphical representation. The evaluated measured variables were examined for their normal distributional properties using Q-Q plots. The line graphs of Figures 4, 5, 6, 7 are based on the descriptive analysis of the frequency of occurrence of each category.

Because of the hierarchical, two-level data structure and the existence of age-specific vocalization types, generalized linear mixed models for the dichotomous variable “melody complexity” were used for crying (C)/non-crying (NC) across the first 6 months and for marginal babbling (BM)/canonical babbling (BC) between 7 and 12 months of life. The value “0” corresponded to vocalizations with a single-arc melody structure, while “1” was used for those with a complex melody structure. Age in months as a metric predictor and the respective vocalization type as a categorical predictor entered the linear multilevel model as fixed effects. The Akaike information criterion (AIC) was used to assess or compare different models and to consider the best model fit. All statistical analyses were performed using SPSS version 26 (IBM Corp., Armonk, NY, USA).

## Results

Cry and non-cry vocalizations exhibiting simple, single-arc melodies dominated during the first month. The relative frequency of occurrence of complex melodies increased thereafter. The turning point toward a clear prevailing of complex structures in cry and non-cry vocalizations was observed in the third month of life (Fig. 4). A permanent higher percentage of complex melody contours was found in the vocal repertoire from the third up to the 12th month of life and was thus also consistently observed in marginal and canonical babbling.

Analysis of complexity degree in the same repertoire yielded a relative frequency of occurrence of 39% vocalizations with single arcs and 60% complex structures across the whole observation period. Complex contours were found to comprise 21% double-arc melodies, 7% triple-arc melodies, 3% of melodies consisting of four and more arcs, and 30% segmented melodies.

A more detailed analysis of melody analysis adjusted to age and vocalization type was yielded by generalized mixed models to the dichotomous variable melody complexity to all 5,808 cry/non-cry vocalizations of the first 6 months of life. The final model revealed a significant positive age effect across the first 6 months of life (*p* = 0.018), while no significant difference was observed between the two vocalization types (*p* = 0.594). The AIC value was 25,307.565, the estimator for random effects was 0.276 (*p* = 0.04).

A corresponding generalized mixed model was calculated based on the 3,429 signals of marginal and canonical babbling across the 7th−12th month of life. No age effect was observed (*p* = 0.293), but a significant higher number of complex structures were found in canonical babbling compared to marginal babbling during this age period (*p* < 0.001). The AIC value was 14,964.803, the estimator for random effects was 0.006 (*p* = 0.528).

**Fig. 8:**
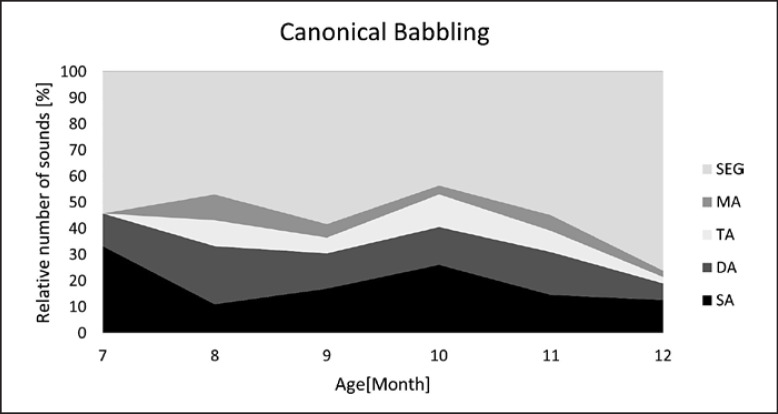
Developmental profile of melody structure in canonical babbling. Melody structures: SA, single-arc melodies; DA, double-arc melodies; TA, triple-arc melodies; MA, multiple-arc melodies, >3 arcs; SEG, segmentations.

Developmental profiles for each vocalization type are visualized in Figures 5, 6, 7, 8. Note the different age intervals of the diagrams.

## Discussion

The data across the first 6 months of life showed a significant increase in complex melody structures in both natural crying and the non-cry repertoire. Beginning at about 35–50% of complex melodies, the number increased to about 70% over the next months. This supports the assumption of a systematic increase in complexity as found in cross-sectional analysis [12, 13]. Regarding the developmental profiles, the increase in complexity is even more evident in the NC repertoire of the first 6 months. Although the developmental profiles seemed to demonstrate slight differences, no statistically significant differences in complex structures were found between cry and non-cry vocalizations across the first 6 months when applying a generalized linear mixed model.

Regarding subgroup shares (DA, TA, MA, SEG), which reflect the degree of melody complexity, there were also marked similarities between the C and NC repertoire (Fig. 5, 6): in both vocalization types, segmented melodies (SEG) predominated among the complex structures. This buttresses the hypothesis of the importance of inner-melodic pauses as part of the very early articulatory exercises according to the laryngeal articulator model introduced by Esling (2005) [32, 33]. This model postulates that articulatory development begins with laryngeally constricted settings, namely, due to sphinctering of the aryepiglottic folds. This leads to breaks within the melody contour, which can be either complete or incomplete, the latter causing creaky or straw bass elements [30]. In a previous study, the existence of those constriction phenomena was investigated, and their regular occurrence observed in C and NC repertoire over the first months [31]. Here, the age-dependent occurrence of these phenomena also demonstrated their increase with ongoing articulatory development in both vocalization types. However, as vocal development progresses, the observed segmentations of melody are no longer primarily due to laryngeal constrictions, which decrease with age [31], but to rhythmicity through syllabification. This was particularly evident in their high proportion among the complex structures in the diagram of canonical babblers (Fig. 8).

The second most common category of complex structures were melodies consisting of two combined single arcs to generate a double-arc melody (DA). The DA share was about 15–20% in C and NC over the first 6 months of life. The high share compared to other unsegmented melodies (TA, MA) may have two reasons: (1) a doubling of basic building blocks is the simplest strategy in generating complexity, so one could expect this stage to occur frequently, (2) a high occurrence of double-arc melodies in pre-speech utterances could point to their significance for the acquisition of disyllabic words [34, 35, 36]. It was particularly interesting that the high DA share was typical for NC during the first months of life and for marginal babbling across 7–12 months, while TA and MA structures occurred comparatively less frequent (cf. Fig. 6, 7).

From month 7–12, only marginal (BM) and canonical (BC) babbling, the typical non-cry vocalizations at this age, were investigated. For vocal development, the natural crying is hardly or no longer relevant. Besides, it is more difficult to record crying at this age. The most striking finding in the BM development diagram was the high stability in the frequency of occurrence of all types of melody structure. The proportion of complex structures overall was between 55 and 65% and showed a slight cyclical movement in all categories (Fig. 7). This variability is interpreted to demonstrate the scaffolding function of melody as postulated by the MD model and the observed cyclic fluctuations to reflect the dynamic interaction between phonatory and articulatory activity [12, 28]. Again, further research is needed to shed light on the direct relationship between melody production and articulation at the vocalization level.

While the share of complex structures in marginal babbling did not increase further after the sixth month of life, a significant increase of complex structures was found in canonical babbling during the same age period (Fig. 7, 8). Complexity gained values of about 90% and was significantly higher compared to that measured in marginal babbling. Segmentations related to syllables were the most frequent complex structures typical for canonical babbling.

Two observations were particularly striking at 7–12 months of age: while all categories of melody structure, i.e., SA, DA, TA, MA, SEG, that were already present in early natural crying can still be found now, the use of individual melody structures in sound production depends on vocalization type. Besides reflecting continuity and complexity increase in melody development across the first 12 months, this also demonstrates an intentional, flexible “usage” of melody under ongoing maturation of vocal tract function.

In summary, the observations underline the development from simple melodies to complex melodies that provides constituents (building blocks) for producing syllables, words, and sentences later on. Furthermore, the findings indicate the importance of melody to move from expressions of emotion to meaning, to move from emotive sounds to abstract words [10, 11, 12].

## Conclusion

The study demonstrated that human infants are able to communicate with melodic patterns long before vocabulary and grammar are established. This provides further evidence of the preparatory function of early melody development for subsequent steps of language development. Increase in melody complexity also reflects increasing vocal control as well as vocal flexibility in all vocalization types, including natural crying, during the first year of life. To produce variable melody structures with tiny vocal folds requires a well-functioning and very responsive laryngeal-respiratory coordination under a steady maturation without marked discontinuity. This is a good prerequisite for approaches to use melody complexity as a risk marker for a reduced vocal control ability.

## Statement of Ethics

An ethics statement is not applicable because this study is based exclusively on anonymized data. The audio files analyzed here were selected from the anonymized archive of the Centre for Prespeech Development and Developmental Disorders, University Hospital, Würzburg, Germany. Therefore, ethical approval was not required. Original recordings were approved by the respective ethical board and were carried out in accordance with relevant guidelines and regulations to guarantee that the research was conducted ethically in accordance with the World Medical Association Declaration of Helsinki; informed consent signed by parents was given.

## Conflict of Interest Statement

The authors have no conflicts of interest to declare.

## Funding Sources

There are no funding sources to declare.

## Author Contributions

All authors have made significant contributions to the conception of the paper. K.W. and T.K. designed the research paper. K.W. supervised and coordinated the recordings in several of their earlier projects and supervised the analyses. T.K. and M.W. analyzed the melodies, identified the different vocalization types, and measured the different melody structures. T.K. and M.W. wrote the main text together with K.W. T.K. and M.W. together performed the statistical analysis. All authors jointly interpreted the results and edited the paper.

## Data Availability Statement

Data are not publicly available on legal grounds. All data generated or analyzed during this study are included in this article. Further inquiries can be directed to the corresponding author.

## Figures and Tables

**Table 1:** Number of vocalizations analyzed for each subject per month

Infant	Sex	Age, months			Total
		1	2	3	4	5	6	7	8	9	10	11	12	
1	Male	47	7	92	71	199	44	25		37	2			524
2	Female	84	103	112	198	11	18	12		21	16			575
3	Female	79	82	151	138	21	94	74	15	20	26	22		722
4	Female	8	134	158	141	200	99				54	123		917
5	Female	81	87	105	124	104	85		81	60	76	136		939
6	Male	104	200	295	266	149	127	54	40	74	92	56		1,457
7	Female		149			68	150	68	97	152	122	72		878
8	Male	49	267	24					10	141	30	168	37	726
9	Female	64	139			196	35	40	121	141	163	33	153	1,085
10	Male	144	148			133	224	93	174	120	56	199	123	1,414
Total		660	1,316	937	938	1,081	876	366	538	766	637	809	313	9,237
